# Psychometric properties of the quality of life in short statured youth (QoLISSY) questionnaire within the course of growth hormone treatment

**DOI:** 10.1186/s12955-019-1118-9

**Published:** 2019-03-18

**Authors:** Janika Bloemeke, Neuza Silva, Monika Bullinger, Stefanie Witt, Helmuth-Günther Dörr, Julia Quitmann

**Affiliations:** 1 Center for Psychosocial Medicine, Institute for Medical Psychology, University Medical Center Hamburg-Eppendorf in Hamburg, Hamburg, Germany; 2 Center for Research in Neuropsychology and Cognitive and Behavioral Intervention (CINEICC), Faculty of Psychology and Education Sciences, University of Coimbra, Coimbra, Portugal; 3 Clinic for Children and Adolescents, University Hospital Erlangen-Nurnberg, Erlangen, Germany

**Keywords:** Health-related quality of life, Patient-reported outcome, Idiopathic growth hormone deficiency, Small for gestational age, Idiopathic short stature, Human growth hormone treatment

## Abstract

**Background:**

The Quality of Life of Short Stature Youth (QoLISSY) questionnaire is a patient- and parent-reported outcome measure assessing health-related quality of life (HRQOL) in short stature youth. This study evaluates the psychometric properties of the QoLISSY questionnaire within a German prospective trial of short statured children treated with human growth hormone (hGH).

**Method:**

The instrument was administered to children with idiopathic growth hormone Deficiency (IGHD) and small for gestational age (SGA) before and after 12 month of hGH treatment. Children with idiopathic short stature (ISS) served as a reference group receiving no treatment. Psychometric testing included scale distribution characteristics, reliability (internal consistency), criterion-and convergent validity (correlations with the generic KIDSCREEN-Index, inter-correlations among QOLISSY subscales), known-group validity (treatment status, height SDS), and responsiveness analysis (ability to detect change).

**Results:**

One hundred fifty-two parents and 66 children/adolescents completed both HRQOL assessments. The QoLISSY demonstrated good reliability with Cronbach’s alpha > .70. Moderate significant correlations between QoLISSY domains and the KIDSCREEN-10 Index supported criterion validity. Statistically significant differences in HRQOL were observed between treatment groups at baseline with children who were about to start treatment reporting a significantly lower HRQOL compared to the children who will not receive treatment. No significant differences were found between the level of short stature based on height SDS scores (≤ − 2 SDS, > − 2 SDS). Furthermore, the instrument detected significant changes in HRQOL between the treated and the untreated group in patient-reports.

**Conclusions:**

In conclusion, the scales showed satisfactory reliability, adequate validity and ability to detect change in self-reported HRQOL within GH treatment. Findings support QoLISSY’s further use in clinical trials, offering the opportunity to adequately assess HRQOL from the patients’ and caregivers’ perspective to improve patient-centered care.

## Background

In recent years, health-related quality of life (HRQOL) has become an important health outcome indicator for use in clinical trials as well as in epidemiological analysis and health service research. Furthermore, it received increased recognition as a relevant health indicator in children with chronic conditions [1]. Using patient-reported outcome (PRO) instruments to assess HRQOL can highlight patients’ unmet needs, improve communication between clinicians and patients, capture the individual and societal impact of a disease, and provide additional information about intervention outcomes directly from the patient’s perspective. Hence, HRQOL instruments are being increasingly used in adults and children to assess information about the subjective perception of health and to evaluate the effects of treatments from the patients’ and caregivers’ perspectives [2–7].

The multidimensional concept of HRQOL describes the subjective perception of health status and includes physical, social, emotional and mental domains of wellbeing and functioning [2, 8]. In addition to generic HRQOL instruments, covering the full range from excellent to poor health, disease-specific measures are necessary to capture the burden and experience of defined health conditions [9–12]. Particularly when assessing HRQOL in chronic and rare diseases, such as short stature in youth, generic PRO measures might be not sensitive enough to detect aspects of diagnosis and treatment that affect the patients’ HRQOL, underlining the need for disease-specific measures [2, 7]. Assessments of HRQOL in short stature youth have been conducted using both generic and disease-specific instruments such as the PedsQL Generic Core Scales [13, 14] and the disease-specific Quality of Life of Short Stature Youth (QoLISSY) questionnaire [3, 12, 15]. Although research has shown that using a generic HRQOL instrument in short stature children can detect differences in HRQOL and psychosocial functioning when compared to children with normal stature [13, 14, 16], it has been shown that short stature-specific instruments can detect changes in HRQOL in children who received treatment, when compared to untreated children, while generic instruments were not sensitive enough to detect these changes [17]. This finding underlines the importance of assessing HRQOL in longitudinal studies with disease-specific instruments, because if significant differences in HRQOL are no longer detected at least one year after children start treatment, then a positive effect of treatment on HRQOL can be reasonably assumed.

Short stature is defined as a height being more than 2 standard deviations (SD) below the mean height of the corresponding population [18, 19] and has been associated with lower HRQOL [20, 21]. In order to improve patients’ height, the short statured children can be treated with human growth hormone (hGH) within the accepted medical indications [22–24]. According to the European Medicine Agency (EMA) this treatment option is only available for defined diagnoses, including idiopathic growth hormone deficiency (IGHD) and being born small for gestational age (SGA), but not for children with idiopathic short stature (ISS) [25]. Primary clinical outcomes of hGH treatment such as the increase in height have been complemented by HRQOL as a relevant endpoint [26]. Results from studies examining the effects of hGH treatment on HRQOL are contradictory; some studies report an increase in HRQOL due to treatment [17, 27], while others report no changes in HRQOL [28]. Furthermore, the use of different assessment approaches (generic and disease-specific) within these studies makes a comparison quite challenging [29]. When evaluating the change of HRQOL in intervention studies, the responsiveness (ability to detect change) of HRQOL instruments is an important characteristic.

To examine if the condition-specific QoLISSY questionnaire is a valid, reliable and responsive disease-specific instrument that measures HRQOL of short stature youth in a longitudinal trial setting and can be used as a health outcome indicator of hGH interventions, this study aimed to evaluate the psychometric performance of the QoLISSY questionnaire within a prospective observational study. Thus, reliability, validity and responsiveness to change were investigated in a sample of children and adolescents with IGHD and SGA at start and within 1-year after hGH treatment, documenting HRQOL in untreated children/adolescents with ISS for comparison purposes.

## Methods

### Participants and study procedure

After receiving ethical approval from the respective ethics committees of the participating centers (medical chambers of Hamburg, Saxon, Hessen, Nordrhein and the ethics committees of the University Hospitals of Erlangen – Nürnberg, Cologne, Magdeburg and Munich), eleven pediatric endocrinologists from various children hospitals and medical practices in Germany agreed to recruit patients for the present study. Included were newly diagnosed children and adolescents and their parents with IGHD and SGA before hGH treatment was initiated. Children diagnosed with ISS served as a reference group in this study. Children aged 8–12 years and adolescents with a late diagnosis > 13 years were invited to complete HRQOL instruments. For the parent-report, parents of children, 8–12 years and > 13 years were recruited for the study as well as parents of younger children aged 4–7 years. Participants were excluded from the study if they were diagnosed with any other condition that results in short stature (e.g. skeletal dysplasia, chromosomal abnormalities, etc.), if they did not meet the respective age groups, if they were cognitively impaired or had a lack of adequate linguistic competency to complete HRQOL assessments. Informed consent from all participants was obtained before the start of the study. Sample size calculations, using the Software PASS 2008, suggested that with a power of 80% to detect changes in the QoLISSY *total score* (sum of the scales *physical*, *social* and *emotional*) and including drop outs in the calculations, the desired sample size should include each *N* = 160 children/adolescents in the intervention and control group and *N* = 240 parents. Children in the intervention group (diagnosed with SGA or IGHD) were treated with daily subcutaneous injections with hGH, whereas children in the reference group (diagnosed with ISS) received no treatment.

The parents and their children were asked to provide HRQOL assessments before the start of hGH treatment (baseline, T0) and at 12 month after the start of treatment (T1). The questionnaires were handed out to the participants in the respective center and were completed on-site at both points of measurement. Thereafter, the staff sent the questionnaires by post to the study center in Hamburg. In addition to HRQOL questionnaires, sociodemographic and clinical data such as age, gender body weight and height were assessed by a clinician at both timepoints. Height standard deviation scores (SDS) was calculated with the LMS formula [30] based on reference data by Kromeyer-Hausschild [31]. Children diagnosed with ISS not undergoing hGH treatment followed the same procedure.

### Measures

HRQOL was assessed with the generic KIDSCREEN-10 index and with the condition-specific QoLISSY questionnaire.

The QoLISSY questionnaire was developed in accordance with the guidance on PRO instrument development of the US Food and Drug Administration [32] within a cross cultural study aiming to assess HRQOL in children and adolescents with ISS and IGHD. The questionnaire is available in self-report for short statured children and adolescents aged 8–18 years and for parents of children aged 4–18 years (observer-report). The instrument assesses HRQOL in the core domains *physical* (6 items), *social* (8 items) and *emotional* (8 items)*,* which are summed up in the *total score* (22 items). Additionally, three domains measure predictors of quality of life (*coping*, 10 items; *beliefs*, 4 items *and treatment*, 14 items) in child- and parent-report. The parent’s version contains two supplementary domains that refer to the parent’s worries about their child’s future (*future*, 5 items) and the impact of the child’s condition on the parents’ wellbeing (*effects on parents*, 11 items). The response scale consists of a five-point Likert scale ranging from “not at all/never” to “extremely/always”. Psychometric results from the original QoLISSY study prove satisfactory reliability with Cronbach’s ranging from *α* = .82 (*coping* scale) to *α* = .92 (*total QoL score*) in self-report and from *α* = .86 (*physical* scale) to *α* = .95 (*total QoL score*) in parent-report [33]. Psychometric performance of the QoLISSY questionnaire has also been proven to be satisfactory in further cross-sectional validation studies in the US, Italy, Belgium and in the Netherlands [34–37]. At present, QoLISSY has also been validated for use in children being born small for gestational age (SGA) and achondroplasia [38, 39].

The KIDSCREEN-10 index is a unidimensional generic measure to assess the HRQOL in children and adolescents (8–18 years) from the child- and parent perspective on a five-point Likert scale. Psychometric properties of the index as assessed in a prior study with a sample of children and adolescents within the original QoLISSY study were satisfactory in self - and parent-reports with good validity and reliability (Cronbach’s alpha in child-report *α* = .81, proxy-report *α* = .80) [40, 41].

### Statistical analysis

All data analyses were conducted using the Statistical Package for the Social Sciences, v.21 [42]. The significance level was set at *p* < .05 and all assessed data of the participants was pseudonymized for the analysis. First, descriptive statistics were calculated for sociodemographic and clinical variables. The homogeneity of sample characteristics across the diagnostic groups at baseline was examined by *χ*^*2*^ tests (categorical variables) or independent-samples analysis of variance (continuous variables).

Raw QoLISSY scores were transformed into 0 to 100 scores with higher values representing higher HRQOL. The QoLISSY *total score* was calculated by summing up the core domains *physical*, *social* and *emotional*. Mean scale scores were computed and missing data that were random and less than 20% of the values were replaced with the individual mean score for each variable.

Descriptive QoLISSY statistics were calculated on a scale level at baseline (T0) and after 12 month after the onset of hGH treatment (T1) for child- and parent-report including mean (*M*), standard deviation (*SD*), skewness, kurtosis and floor/ceiling effects. Floor and ceiling effects were considered to be present if more than 15% of respondents achieved the highest or lowest possible score, respectively [43].

To evaluate the reliability of the QoLISSY scales, Cronbach’s alpha coefficients (*α*) were calculated for each scale at baseline and at T1 for both patient- and parent reports, considering *α* > .70 as an indicator of good internal consistency [44].

The inter-rater reliability was examined at the individual and the group levels [45], by using, respectively, intraclass correlation coefficients (ICC; two-way mixed model, absolute agreement, 95% confidence interval [CI]) and multivariate analyses of covariance (MANCOVA) for repeated measures, entering the rater (parent vs. child) as the within-subject factor and children’s age, gender, height deviation group and treatment status (only at T1) as covariates. ICC reference values were: *ICC* < .40 as poor agreement, *ICC* between .41 and .60 as moderate agreement, *ICC* between .61 and .80 as good agreement, and *ICC* > .81 as excellent agreement [46].

The criterion validity was examined by calculating Pearson correlation coefficients (*r*) between the QoLISSY scales and the KIDSCREEN-10 Index at both points of measurement for the child- and parent-report. Although the KIDSCREEN instruments can be considered as gold standard instruments to assess HRQOL in children and adolescents, they measure HRQOL at a generic level, while the QoLISSY questionnaire assesses HRQOL at a disease-specific level; therefore, moderate positive correlations (*r* > .30) were considered indicators of good criterion validity [44]. To assess convergent validity as part of the construct validity, inter-correlations between the QoLISSY scales were calculated using Pearson’s correlation coefficient as well.

To examine known-groups validity, multivariate analyses of covariance (MANCOVA) were performed at both measurement points for the child- and parent-report to compare the HRQOL dimensions across the treatment status (children with IGHD or SGA who were treated vs. untreated children with ISS) and height deviation groups of normal stature (> − 2 *SDS*) vs. short stature (≤ − 2 *SDS*), while controlling for children’s age. For the QoLISSY *total score* and when multivariate effects were significant, univariate analyses of covariance (ANCOVA) were performed to examine which QoLISSY scales were significantly different between groups.

Finally, to test the responsiveness of the QoLISSY questionnaires to detect changes in HRQOL dimensions within the course of hGH treatment, a repeated measures MANCOVA was performed. The measurement points (baseline vs. T1) were entered as the within-subjects factor, while the treatment status (children with IGHD or SGA who were treated vs. untreated children with ISS) as the between-subjects factor, and children’s age and time difference between both measurement points as covariates. A repeated measures ANCOVA was performed for the QoLISSY *total score* and for the generic KIDSCREEN-10 Index, respectively. Furthermore, a repeated measures MANCOVA testing the HRQOL changes from baseline and 1-year follow-up (T1) between patients who reached normal height and patients with current short stature at T1 was calculated. Children’s age and time difference between both measurement points were entered as covariates. Presented effect-size measures were analyzed based on Cohen (1988), considering a small effect when ŋ_p_^2^ ≥ .01, a medium effect when ŋ_p_^2^ ≥ .06, and a large effect when ŋ_p_^2^ ≥ .14 [47].

In addition, Pearson’s correlation analysis between height SDS increase (i.e, height SDS at T1 – height SDS at baseline) and the change in the QoLISSY *total score* (i.e., QoLISSY *total score* at T1 – QoLISSY total score at baseline) were performed with child and parent data to determine the relationship between height change and HRQOL change.

## Results

### Sample description

A total of 154 participants were recruited at baseline, with 66 children/adolescents and 152 parents completing both HRQOL assessments. Of these and with a drop-out rate of 15.6% (*N* = 24), 130 participants also completed the HRQOL assessments at T1 with 70 patient-reports and 126 parent-reports available. Since patients grow older during the study process, more patient-reports were available at T1. The average time frame between the baseline assessment and T1 ranged from 6 to 18 month (*M* = 12.54, *SD* = 2.2). Clinical and sociodemographic sample characteristics of the participants at both measurement points are presented in Table 1. At baseline, SGA patients were significantly younger than patients diagnosed with IGHD or ISS (*F*_(2)_ = 9.21, *p* < .01). This trend was continued at T1 with SGA patients being significantly younger than patients diagnosed with ISS (*F*_(2)_ = 6.74, *p* < .01). Furthermore, SGA patients were significantly smaller at baseline (*F*_(2)_ = 12.74, *p* < .01) and at T1 (*F*_(2)_ = 5.59, *p* < .01) compared to patients with IGHD or ISS. With regard to height SDS, patients with SGA and IGHD were significantly the smallest compared to patients with ISS at baseline (*F*_(2)_ = 8.70, *p* < .01). Moreover, a significantly higher percentage of patients with ISS had already reached a normal height by definition at baseline (*SDS* > − 2), compared to children/adolescents with IGHD or SGA before treatment (*χ*_*(2)*_^*2*^ = 13.92, *p* < .01). At T1 there were no significant differences in height SDS (*F*_(2)_ = 7.58, *p* = .47) or in height deviations groups (*χ*_*(2)*_^*2*^ = .85, *p* = .65) across diagnoses. Also, there were no significant differences in gender (*χ*_*(2)*_^*2*^ = 5.93, *p* = .052) or treatment length (*F*_(2)_ = .516, *p* = .59) across diagnoses groups.

**Table 1:** Sample characteristics at baseline (T0) and after 1 year of hGH treatment (T1)

	T0 (*N* = 154)			T1 (*N* = 130)		
	IGHD (*N* = 65)	SGA (*N* = 58)	ISS (*N* = 31)	IGHD (*N* = 60)	SGA (*N* = 48)	ISS (*N* = 22)
Age (yrs.) M (SD)^a,b^	8.09 (3.34)	6.55 (2.64)	9.45 (3.49)	9.22 (3.37)	7.81 (2.76)	10.68 (3.24)
*Missing, n (%)*	–	–	–	1 (1.7)	–	–
Age group, N (%)						
4–7 years	34 (52.3)	41 (70.7)	7 (22.6)	26 (43.3)	28 (58.3)	4 (18.2)
8–12 years	22 (33.8)	15 (25.9)	16 (51.6)	22 (36.7)	18 (37.5)	10 (45.5)
13–17 years	9 (13.8)	2 (3.4)	8 (25.8)	11 (18.3)	2 (4.2)	8 (36.4)
*Missing (%)*	–	–	–	1 (1.7)	–	–
Gender, N (%)						
Male	48 (73.8)	33 (56.9)	16 (51.6)	44 (73.3)	29 (60.4)	11 (50.0)
Female	17 (26.2)	25 (43.1)	15 (48.4)	16 (26.7)	19 (39.6)	11 (50.0)
Height (cm), M (SD)^c^	117.11 (17.44)	108.01 (13.45)	126.24 (18.86)	127.04 (17.53)	118.89 (13.65)	132.48 (17.77)
*Missing (%)*	–	1 (1.7)	1 (3.2)	2 (3.3)	2 (4.2)	1 (4.5)
Height deviation (SDS), M (SD)^d^	− 2.61 (.61)	−2.65 (0.63)	− 2.11 (.51)	− 1.91 (.64)	−2.05 (.67)	− 2.06 (.73)
*Missing (%)*	1 (1.5)	1 (1.7)	1 (3.2)	2 (3.3)	5 (10.4)	1 (4.5)
Height deviation group, N (%)^e^						
Normal height (< − 2 *SDS*)	11 (16.9)**	6 (10.3)**	13 (41.9)**	32 (53.3)	23 (47.9)	10 (45.5)
Short stature (≥ − 2 *SDS*)	54 (83.1)	51 (87.9)	17 (54.8)	25 (41.7)	23 (47.9)	11 (50.0)
*Missing (%)*	–	1 (1.7)	1 (3.2)	3 (5.0)	2 (4.2)	1 (4.5)
Treatment length (months), M (SD)	–	–	–	12.64 (1.93)	12.25 (2.43)	12.73 (2.76)
*Missing (%)*	–	–	–	1 (1.7)	–	–

Note: *M =* Mean, *SD* = Standard deviation, *SDS*=Standard deviation score, IGHD = idiopathic growth hormone deficiency, SGA = short for gestational age, ISS = idiopathic short stature, T0 = baseline, T1 = 12 month after start of hGH treatment

^a^ children with SGA were significantly younger than children with IGHD or ISS at baseline (*F*_(2)_ = 9.21, *p* < .01)

^b^ children with SGA were significantly younger than children with ISS at T1 (F_(2)_ = 6.74, p < .01)

^c^ children with SGA were significantly smaller than children with ISS or IGHD at baseline (F_(2)_ = 12.74, p < .01) and at T1 (F_(2)_ = 5.59, p < .01)

^d^ children with SGA and IGHD were significantly the smallest compared to children with ISS at baseline (*F*_(2)_ = 8.70, *p* < .01)

^e^ a significant higher percentage of children with ISS already reached a normal height at T0 (χ_(2)_^2^ = 13.92, p < .01)

* *p* < .05; ** p < .01, two-tailed

### Descriptive statistics and reliability

Most QoLISSY dimensions showed a slight skew to the right, indicating a predisposition for high HRQOL (ranging from 0 to 100, with higher values representing higher HRQOL) in child- and parent-report at baseline and T1 across all diagnoses groups. However, the parent-reported *coping* and *effects on parents* scale at baseline and the parent-reported *treatment* scale at T1 as well as the child-reported *beliefs* scale at T1, showed a slight skew to the left, indicating lower HRQOL. The Cronbach’s alpha values were higher than α > .70, except for the child-reported *coping* scale at T1 (α = .65), indicating good internal consistency for raters and assessment points. Highest consistency was found in the *total score* (α > .90). Considering the threshold of 15% of respondents scoring at the lowest or highest possible categories, no ceiling or floor effects were found for any of the patient- or parent-reported QoLISSY scales (Table 2).

**Table 2:** Descriptive statistics and reliability for the QoLISSY scales across all diagnoses

		Descriptive Statistics							Reliability	
Patient-report		*N*	*M*	*SD*	Skewness	Kurtosis	Floor (%)	Ceiling (%)	*N*	α
Physical	T0	65	55.96	26.66	−.32	−.82	4.6	.00	65	.83
	T1	70	61.50	25.36	−.48	−.63	.00	.00	64	.83
Social	T0	66	52.94	26.84	−.20	−.91	4.5	.00	62	.87
	T1	70	62.15	23.88	−.72	−.17	1.4	.00	62	.82
Emotional	T0	65	53.41	27.00	−.06	−1.18	.00	1.5	58	.88
	T1	69	62.49	24.65	−.61	−.27	1.4	1.4	66	.87
Coping	T0	66	62.87	21.95	−.47	−.65	.00	.00	65	.82
	T1	66	58.81	16.45	.02	−1.07	.00	1.5	63	.65
Beliefs	T0	66	49.62	32.07	−.10	−1.21	10.6	4.5	66	.88
	T1	68	51.56	31.75	.09	−1.20	8.8	8.8	68	.86
Treatment	T0	–	–	–	–	–	–	–	–	–
	T1	50	60.52	17.64	−.49	−.33	.00	.00	44	.83
Total Score^a^	T0	64	54.11	24.96	−.24	−.89	.00	.00	54	.95
	T1	69	61.82	22.87	−.60	−.36	.00	.00	54	.94
Parent-report		*N*	*M*	*SD*	Skewness	Kurtosis	Floor (%)	Ceiling (%)	*N*	α
Physical	T0	150	52.57	26.74	−.09	−1.06	1.3	1.3	146	.88
	T1	126	58.12	25.64	−.23	−1.19	.00	2.4	117	.89
Social	T0	150	51.43	24.94	−.05	−.94	2.7	.00	129	.86
	T1	125	57.25	25.68	−.35	−.86	1.6	0.8	113	.87
Emotional	T0	150	52.34	25.95	−.06	−.87	1.3	2.0	143	.89
	T1	125	58.25	24.65	−.27	−.68	.80	2.4	116	.87
Coping	T0	135	50.97	17.86	.20	−.47	.00	.00	125	.75
	T1	115	51.08	18.00	−.09	−.45	.00	1.7	109	.78
Beliefs	T0	144	54.99	29.41	−.12	−1.05	6.3	9.0	142	.88
	T1	123	55.95	30.10	−.16	−1.08	4.9	10.6	123	.88
Treatment	T0	–	–	–	–	–	–	–	–	–
	T1	95	59.76	18.90	.33	−.62	.00	.00	81	.87
Future	T0	140	58.43	30.31	−.18	−1.07	4.3	12.9	140	.88
	T1	119	62.17	29.09	−.46	−.89	3.4	11.8	111	.88
Effects on parents	T0	148	52.54	22.88	.24	−.75	.00	0.7	139	.86
	T1	125	58.37	23.04	−.15	−.65	.00	2.4	112	.86
Total Score^a^	T0	147	52.06	23.73	.06	−.92	.00	.00	123	.94
	T1	125	57.96	23.54	−.28	−.84	.00	.00	103	.95

Note: *M* = Mean, *SD* = Standard deviation, α = Cronbach’s alpha value, T0 = baseline, T1 = 12 month after start of hGH treatment

^a^ Sum of the scales physical, social, emotional

Regarding inter-rater reliability, the ICC values indicated moderate parent-child agreement, except for the coping scale and treatment subscale where a higher disagreement of HRQOL assessment between children and parents was found. At a group level, the MANCOVA for repeated measures showed no significant multivariate differences between raters at baseline, Pillai’s trace = .11, *F*_(5, 47)_ = 1.19, *p* = .33, ŋ_p_^2^ = .11, and at T1, Pillai’s trace = .30, *F*_(6, 30)_ = 2.10, *p* = .08, ŋ_p_^2^ = .30. The univariate effects are presented in Table 3. In addition, no significant multivariate effects of the interaction between rater and children’s sex at baseline (Pillai’s trace = .05, *F*_(5, 47)_ = 0.44, *p* = .82, ŋ_p_^2^ = .05) and T1 (Pillai’s trace = .32, *F*_(6, 30)_ = 2.30, *p* = .06, ŋ_p_^2^ = .32), children’s age at baseline (Pillai’s trace = .12, *F*_(5, 47)_ = 1.33, *p* = .27, ŋ_p_^2^ = .12) and T1 (Pillai’s trace = .30, *F*_(6, 30)_ = 2.10, *p* = .08, ŋ_p_^2^ = .30), height deviation group at baseline (Pillai’s trace = .02, *F*_(5, 47)_ = .21, *p* = .96, ŋ_p_^2^ = .02) and at T1 (Pillai’s trace = .20, *F*_(6, 30)_ = 1.24, *p* = .31, ŋ_p_^2^ = .20), or treatment status (T1), Pillai’s trace = .24, *F*_(6, 30)_ = 1.61, *p* = .18, ŋ_p_^2^ = .24, were found.

**Table 3:** Inter-rater reliability: Intraclass Correlation Coefficients and ANCOVA for repeated measures

		Patient-Reports (*N* = 55)	Parent-reports (N = 55)	ANCOVA for repeated measures			ICC [CI]
		*M (SD)*	*M (SD)*	*F*	*p*	Ŋ_p_^2^	
Physical	T0	56.74 (26.59)	48.94 (24.71)	2.36	.13	.04	.56** [.36–.71]
	T1	59.60 (25.93)	51.10 (24.86)	.01	.93	.00	.44** [.23–.62]
Social	T0	54.27 (26.65)	43.32 (23.57)	2.91	.09	.05	.74** [.60–.83]
	T1	62.59 (24.75)	48.38 (26.62)	.22	.64	.01	.44** [.22–.61]
Emotional	T0	53.76 (26.79)	41.88 (22.36)	.36	.55	.01	.67** [.50–.78]
	T1	63.49 (23.32)	49.05 (24.73)	.06	.80	.00	.58** [.39–.72]
Coping	T0	62.18 (22.34)	52.42 (18.90)	2.87	.10	.05	.35** [.11–.56]
	T1	60.90 (16.22)	48.81 (14.82)	4.98	.03	.13	.29* [.03–.50]
Beliefs	T0	48.75 (32.00)	46.02 (30.37)	.02	.89	.00	.61** [.44–.75]
	T1	51.25 (32.68)	48.75 (30.40)	.78	.38	.02	.56** [.36–.70]
Treatment	T0	–	–	–	–	–	–
	T1	62.88 (16.99)	60.10 (16.88)	5.38	.03	.13	.45** [.19–.65]
Total Score ^a^	T0	55.91 (24.46)	44.81 (22.12)	2.39	.13	.04	.74** [.60–.83]
	T1	62.58 (22.95)	53.53 (24.03)	.01	.92	.00	.55** [.36–.70]

Note: *M* = Mean, *SD* = Standard deviation, *F*=F-value, *p* = *P*-value, Ŋp2 = partial eta square, ICC=Intraclass correlation coefficient (two-way mixed model, absolute agreement, 95% confidence interval [CI], T0 = baseline, T1 = 12 month after start of hGH treatment

* *p* < .05; ** p < .01, two-tailed

^a^ sum of the scales physical, social, emotional

### Criterion and convergent validity

Table 4 summarizes the correlations between the QoLISSY scales and the KIDSCREEN-10 Index. For patient-reports, moderate positive correlations were found between the disease-specific and generic HRQOL, with coefficients ranging from *r* = .28 (*belief* scale at T1) to *r* = .46 (*coping* scale at T1); for parent-reports, weak to moderate positive correlations were found, with coefficients ranging from *r* = .13 (*belief* scale at T1) to *r* = .41 (*emotional* scale at baseline). Regarding convergent validity as assessed with inter-correlations among the QoLISSY subscales, moderate to strong positive correlations were found across the *physical*, *social*, *emotional* and *beliefs* scales, in both patient- and parent-reports, as well as across the *future* and *effects on parents* scales of the parent-reported version. In both patient- and parent-reports, the *coping* scale was weakly to moderately positively correlated with all QoLISSY scales and with the *total score*, while the *treatment* scale did not correlate significantly with any of the scales nor with the *total score*. However, it needs to be noted, that the *treatment* subscale was only applied in the treated sample including children with IGHD and SGA, and the other QoLISSY subscales are being tested in a more heterogeneous sample, including non-treated patients with ISS.

**Table 4:** Pearson Correlation coefficients between the QoLISSY scales and the KIDSCREEN-10 Index and QoLISSY interscale-correlations

Patient-reports		KIDSCREEN-10	1	2	3	4	5	6		
1. Physical	T0	.41^**^	–							
	T1	.30^*^	–							
2. Social	T0	.38^**^	.84^**^	–						
	T1	.32^**^	.82^**^	–						
3. Emotional	T0	.43^**^	.71^**^	.79^**^	–					
	T1	.30^*^	.75^**^	.82^**^	–					
4. Coping	T0	.42^**^	.29^*^	.28^**^	.46^**^	–				
	T1	.46^**^	.22	.31^*^	.35^**^	–				
5. Beliefs	T0	.38^**^	.61^**^	.65^**^	.81^**^	.30^*^	–			
	T1	.28^*^	.60^**^	.66^**^	.76^**^	.34^**^	–			
6. Treatment	T0	–	–	–	–	–	–	–		
	T1	.43^**^	.14	.02	−.03	.20	−.12	–		
QoLISSY Total Score ^a^	T0	.44^**^	.92^**^	.95^**^	.90^**^	.38^**^	.75^**^	–		
	T1	.33^**^	.92^**^	.94^**^	.92^**^	.33^**^	.73^**^	.05		
Parents-reports		KIDSCREEN-10	1	2	3	4	5	6	7	8
1. Physical	T0	.27^**^	–							
	T1	.28^**^	–							
2. Social	T0	.31^**^	.77^**^	–						
	T1	.35^**^	.81^**^	–						
3. Emotional	T0	.41^**^	.69^**^	.85^**^	–					
	T1	.27^**^	.73^**^	.86^**^	–					
4. Coping	T0	.26^**^	.14	.16	.25^**^	–				
	T1	.23^*^	.26^**^	.28^**^	.35^**^	–				
5. Beliefs	T0	.34^**^	.56^**^	.59^**^	.69^**^	.16	–			
	T1	.13	.60^**^	.62^**^	.67^**^	.24^*^	–			
6. Treatment	T0	–	–	–	–	–	–	–		
	T1	.27^*^	.04	−.02	−.06	.14	−.13	–		
7. Future	T0	.27^**^	.62^**^	.70^**^	.73^**^	.22^*^	.70^**^	–	–	
	T1	.15	.58^**^	.67^**^	.70^**^	.21^*^	.68^**^	−.07	–	
8. Effects on parents	T0	.36^**^	.55^**^	.60^**^	.60^**^	.25^**^	.56^**^	–	.58^**^	–
	T1	.22^*^	.62^**^	.65^**^	.66^**^	.25^**^	.63^**^	−.02	.74^**^	–
QoLISSY Total Score ^a^	T0	.36^**^	.89^**^	.94^**^	.92^**^	.20^*^	.68^**^	–	.73^**^	.63^**^
	T1	.33^**^	.91^**^	.96^**^	.93^**^	.32^**^	.86^**^	−.01	.70^**^	.69^**^

Note: ^*^ *p* < .05; ^**^ *p* < .01, two-tailed, T0 = baseline, T1 = 12 month after start of hGH treatment

^a^ sum of the scales physical, social, emotional

### Known-groups validity

The MANCOVA for patient-reported QoLISSY scales, controlling for children’s age and height deviation group, yielded no significant multivariate effects of treatment status on QoLISSY scales at baseline, Pillai’s trace = .17, *F*_(5, 54)_ = 2.36, *p* = 0.052, ŋ_p_^2^ = .17. However, a significant univariate effect was found in the *physical*, *social* and *emotional* scale, with children who were about to start treatment reporting a significantly lower HRQOL at baseline compared to the ISS children not receiving treatment. A significant univariate effect was also found for the *total score F*_(1, 158)_ = .7.44, *p* = .008, ŋ_p_^2^ = .11 (see Table 5).

**Table 5:** Univariate analyses of covariance between treatment status at baseline (T0)

	Treated (IGHD/SGA)	Untreated (ISS)			
Patient-reports	*M (SD)* (*N* = 40)	*M (SD)* (*N* = 22)	*F*	*p*	Ŋ_p_^2^
Physical	48.33 (28.00)	69.88 (17.86)	8.44	.005	.12
Social	46.99 (29.03)	64.85 (18.86)	4.72	.03	.07
Emotional	47.35 (25.13)	65.76 (26.67)	5.76	.02	.09
Coping	62.50 (21.40)	62.38 (24.30)	.01	.92	.00
Beliefs	43.28 (29.70)	61.36 (32.76)	3.57	.06	.05
Total Score ^a^	47.56 (25.74)	66.83 (18.04)	7.445	.008	.11
Parents-reports	*M (SD)* (*N* = 99)	*M (SD)* (*N* = 28)	*F*	*p*	Ŋ_p_^2^
Physical	47.84 (25.97)	65.47 (24.05)	8.91	.003	.06
Social	47.40 (24.79)	58.72 (23.79)	4.59	.03	.03
Emotional	48.42 (24.63)	57.86 (26.41)	4.69	.03	.03
Coping	49.10 (16.99)	58.22 (19.56)	3.95	.04	.03
Beliefs	52.52 (29.25)	58.25 (33.29)	1.39	.24	.01
Future	53.93 (30.21)	68.57 (26.62)	8.12	.005	.06
Effects on parents	49.61 (22.34)	56.69 (22.05)	1.48	.22	.01
Total Score ^a^	49.91 (23.54)	61.50 (22.58)	7.70	.006	.05

Note: *M* = Mean, *SD* = Standard deviation, *F*=F-value, *p* = P-value, Ŋp2 = partial eta square, IGHD = idiopathic growth hormone deficiency, SGA = short for gestational age, ISS = idiopathic short stature

Children’s age and height deviation group were included as covariates

^a^ sum of the scales physical, social, emotional

Regarding parent-reports a significant multivariate effect of treatment status was found on QoLISSY scales, Pillai’s trace = .12, *F*_(7, 117)_ = 2.31, *p* = 0.03, ŋ_p_^2^ = .12. Subsequent univariate analysis showed significant differences between the treated and the untreated group in the *physical*, *social*, *emotional*, *coping* and *future* domain, with parents of children in the treated group reporting a lower HRQOL in these domains at baseline, compared to parents of children who remained untreated. In addition, a significant effect of treatment status was found in the *total score, F*_(1, 141)_ = 7.70, *p* = .006, ŋ_p_^2^ = .05, with parents of treated children reporting significantly lower HRQOL than parents of children with ISS (*p* < .01) (see Table 5).

After treatment, no significant multivariate effect of treatment status on QoLISSY scales were found in child-report, Pillai’s trace = .07, *F*_(5, 54)_ = .85, *p* = 0.51, ŋ_p_^2^ = .07, or for the *total score*, *F*_(1, 62)_ = .002, *p* = .966, ŋ_p_^2^ = .00. Also the parent-reports yielded no significant multivariate effect of treatment status on QoLISSY scales, Pillai’s trace = .064, *F*_(7, 95)_ = .93, *p* = 0.48, ŋ_p_^2^ = .06. However, a significant effect of the *physical* scale was found in the univariate analysis in the parent-report *F*_(1, 101)_ = 5.094, *p* = .02, ŋ_p_^2^ = .04. The univariate analysis for the *total score* revealed no significant effect, *F*_(1, 114)_ = 3.21, *p* = .07, ŋ_p_^2^ = .02.

Regarding effects of height deviation groups (children/adolescents with a height deviation ≥ − 2 *SD* vs. children/adolescents who already achieved normal height at the time of assessment) at baseline, the MANCOVA, controlling for children’s age and diagnosis (GHD or SGA vs. ISS), showed no significant multivariate effects on the QoLISSY scales for patient-reports, Pillai’s trace = .09, *F*_(5, 54)_ = 1.066, *p* = 0.38, ŋ_p_^2^ = .09, and for parent-reports, Pillai’s trace = .06, *F*_(7, 117)_ = 1.18, *p* = .32, ŋ_p_^2^ = .06. Nevertheless, a significant univariate effect was found in the *social* scale for patient-reports, *F*_(1, 58)_ = 4.89, *p* = .03, ŋ_p_^2^ = .07, and for parent-reports, *F*_(1, 123)_ = 5.55, *p* = .02, ŋ_p_^2^ = .04, with short statured children scoring significantly lower in this domain. The univariate analyses are presented in Table 6. In addition, the univariate ANCOVA for the QoLISSY *total score* showed no significant differences between children/adolescents with current short stature and their peers who achieved normal height for patient-reports, *F*_(1, 58)_ = 3.87, *p* = .06, ŋ_p_^2^ = .06, and for parent-reports, *F*_(1, 141)_ = 3.44, *p* = .06, ŋ_p_^2^ = .02.

**Table 6:** Univariate analyses of covariance between height deviation groups at baseline (T0)

	Short stature (≤ −2 *SDS*)	Normal height (> −2 *SDS*)			
Patient-reports	*M (SD)* (*N* = 47)	*M (SD)* (*N* = 15)	*F*	*p*	Ŋ_p_^2^
Physical	52.48 (26.83)	66.94 (24.47)	3.43	.06	.05
Social	49.11 (27.72)	66.58 (20.79)	4.89	.03	.07
Emotional	51.07 (26.70)	62.71 (26.82)	1.80	.18	.03
Coping	61.70 (21.52)	64.83 (25.15)	.12	.72	.00
Beliefs	46.14 (33.01)	60.83 (25.38)	2.29	.13	.03
Total Score ^a^	50.89 (25.27)	65.41 (21.03)	3.87	.06	.06
Parents-reports	*M (SD)* (*N* = 101)	*M (SD)* (*N* = 26)	*F*	*p*	Ŋ_p_^2^
Physical	49.95 (26.36)	58.65 (26.43)	1.37	.24	.01
Social	47.08 (24.33)	60.85 (24.67)	5.55	.02	.04
Emotional	48.05 (25.07)	60.04 (24.03)	3.74	.06	.03
Coping	49.92 (17.37)	55.76 (19.56)	1.11	.29	.01
Beliefs	52.72 (30.59)	57.93 (28.59)	.52	.46	.00
Future	54.65 (30.08)	66.92 (28.07)	2.59	.11	.02
Effects on parents	50.27 (22.65)	54.68 (21.41)	.52	.47	.00
Total Score ^a^	50.32 (23.42)	60.28 (23.76)	3.44	.06	.02

Note: *M* = Mean, *SD* = Standard deviation, *SDS*=Standard deviation score, *F*=F-value, *p* = *P*-value, Ŋp2 = partial eta square

Children’s age and height deviation group were included as covariates

^a^ sum of the scales physical, social, emotional

After treatment, no significant multivariate effects of height deviation group on QoLISSY scales, Pillai’s trace = .03, *F*_(5, 54)_ = .33, *p* = 0.88, ŋ_p_^2^ = .03, or for the *total score F*_(1, 62)_ = .01, *p* = .91, ŋ_p_^2^ = .00 were found in patient-report. Also the parents-report yielded no significant multivariate effect of height deviation group on QoLISSY scales, Pillai’s trace = .05, *F*_(7, 95)_ = .82, *p* = 0.56, ŋ_p_^2^ = .05, or for the *total score F*_(1, 114)_ = .03, *p* = .85, ŋ_p_^2^ = .00.

### Responsiveness

The repeated measures MANCOVA for the patient-reported QoLISSY scales, controlling for the effects of age and treatment length, yielded no significant multivariate main effects of time, Pillai’s trace = .18, *F*_(5, 43)_ = 1.84, *p* = .13, ŋ_p_^2^ = .18, or treatment status, Pillai’s trace = .13, *F*_(5, 43)_ = 1.33, *p* = .27, ŋ_p_^2^ = .13. However, the multivariate interaction effect between time and treatment status was statistically significant, Pillai’s trace = .27, *F*_(5, 43)_ = 3.24, *p* = .01, ŋ_p_^2^ = .27. The subsequent univariate analyses (Table 7) revealed that treated children/adolescents in the intervention group (diagnoses IGHD or SGA) reported a significant increase of *physical*, *social* and *emotional* HRQOL from baseline assessment throughout one year of treatment, while untreated patients of the control group (diagnosis ISS) reported a decrease in these HRQOL domains over time.

**Table 7:** HRQOL changes from baseline (T0) and 1-year follow-up (T1) between treated and untreated patients

		Treated (IGHD/SGA)	Untreated (ISS)			
Patient-reports		*M (SD)* (*N* = 33)	*M (SD* (*N* = 18)	*F*	*p*	Ŋ_p_^2^
Physical	T0	48.99 (26.96)	69.91 (18.22)	8.51	< .01	.15
	T1	62.65 (22.85)	61.94 (27.57)			
Social	T0	45.67 (26.64)	66.42 (20.42)	14.76	< .01	.24
	T1	62.04 (25.86)	58.51 (23.70)			
Emotional	T0	47.06 (24.86)	70.71 (26.56)	11.68	< .01	.20
	T1	61.24 (23.29)	62.18 (26.80)			
Coping	T0	60.98 (22.29)	67.78 (21.62)	2.44	.13	.05
	T1	60.56 (16.19)	57.08 (18.03)			
Beliefs	T0	41.86 (30.03)	68.06 (31.21)	3.23	.08	.06
	T1	46.59 (33.88)	56.60 (28.72)			
Total score ^a^	T0	48.88 (24.17)	69.01 (19.50)	9.72	< .01	.16
	T1	61.60 (22.88)	60.88 (24.20)			
KIDSCREEN-10 Index	T0	54.80 (12.06)	51.93 (9.10)	1.06	.30	.02
	T1	55.80 (10.58)	55.66 (13.89)			
Parents-reports		*M (SD)* (*N* = 78)	*M (SD)* (*N* = 20)	*F*	*p*	Ŋ_p_^2^
Physical	T0	46.78 (25.54)	62.50 (25.90)	.14	.71	.00
	T1	54.11 (25.04)	63.92 (26.11)			
Social	T0	48.20 (24.64)	58.62 (24.46)	.79	.38	.01
	T1	55.49 (25.73)	58.50 (27.39)			
Emotional	T0	47.88 (24.37)	58.19 (23.58)	.85	.36	.01
	T1	55.89 (24.63)	60.16 (27.56)			
Coping	T0	48.30 (17.03)	59.89 (21.49)	1.56	.22	.02
	T1	50.42 (15.68)	55.18 (24.01)			
Beliefs	T0	51.92 (28.97)	61.25 (32.23)	.24	.62	.00
	T1	50.88 (29.08)	57.19 (36.01)			
Future	T0	52.05 (30.38)	70.25 (25.31)	3.33	.07	.03
	T1	59.73 (28.37)	63.56 (29.28)			
Effects on parents	T0	49.48 (22.10)	59.84 (24.41)	.25	.62	.00
	T1	55.62 (21.70)	58.99 (27.36)			
Total score ^a^	T0	50.56 (23.57)	60.97 (22.46)	1.33	.25	.01
	T1	57.34 (23.31)	60.63 (25.05)			
KIDSCREEN-10 Index	T0	50.90 (10.16)	51.75 (7.84)	2.71	.10	.03
	T1	51.75 (7.84)	48.56 (12.11)			

Note: Analysis: Univariate analyses of covariance. *M* = Mean, *SD* = Standard deviation, *F*=F-value, *p* = *P*-value, Ŋp2 = partial eta square, IGHD = idiopathic growth hormone deficiency, SGA = short for gestational age, ISS = idiopathic short stature, T0 = baseline, T1 = 12 month after start of hGH treatment

^a^ sum of the scales physical, social, emotional

These results of differences between the treated and the untreated sample were supported by the univariate repeated measures ANCOVA for the QoLISSY *total score*, which also revealed a significant interaction effect between time and treatment status, *F*_(1, 50)_ = 9.72, *p* < .01, ŋ_p_^2^ = .16, while controlling for the effects of age at baseline, *F*_(1, 50)_ = .03, *p* = .86, ŋ_p_^2^ = .00, and treatment length, *F*_(1, 50)_ = 3.97, *p* = .05, ŋ_p_^2^ = .07.

Apart from the significant results in patient-reports, the parent-reports revealed no multivariate main effects of treatment status, Pillai’s trace = 0.14, *F*_(7, 88)_ = 2.07, *p* = .06, ŋ_p_^2^ = .14, or interaction effects between time and treatment status, Pillai’s trace = .06, *F*_(7, 88)_ = .85, *p* = .55, ŋ_p_^2^ = .06. But there was a significant multivariate main effect of time, Pillai’s trace = .19, *F*_(7, 88)_ = 2.91, *p* = .01, ŋ_p_^2^ = .19, which revealed a significant improvement from baseline to T1 on the *effects on parents* scale, for both intervention and control groups, *F*_(1, 94)_ = 7.75, *p* < .01, ŋ_p_^2^ = .08. The univariate analyses for the interaction effect are presented in Table 7. Although differences were not statistically significant the results demonstrate a trend that HRQOL scores improved in all QoLISSY domains in the treated sample, while scores decreased in the *social*, *coping*, *beliefs*, *future*, *effects on parents* scale and in the *total score* in the untreated sample.

Also the ANCOVA for the parent-reported QoLISSY *total score* yielded no significant main effects of time, *F*_(1, 118)_ = 1.02, *p* = 0.31, ŋ_p_^2^ = .01, or interaction effects between time and treatment status, *F*_(1, 118)_ = 1.33, *p* = 0.25, ŋ_p_^2^ = .01, while controlling for the effects of age at baseline, *F*_(1, 118)_ = 16.94, *p* < .01, ŋ_p_^2^ = .13, and treatment length, *F*_(1, 118)_ = .83, *p* = .37, ŋ_p_^2^ = .01. Nevertheless, a significant main effect of treatment status was found for the parent-reported QoLISSY *total score*, *F*_(1, 118)_ = 5.83, *p* = .02, ŋ_p_^2^ = .05. Independently of time of assessment, parents of treated children reported a lower HRQOL than parents of untreated children. The KIDSCREEN-10 Index, revealed no significant main effects of the interaction between time and treatment status in child, Pillai’s trace = .02, *F*_(1, 49)_ = 1.06, *p* = .31, ŋ_p_^2^ = .02, and parent-report, Pillai’s trace = .03, *F*_(1, 88)_ = 2.71, *p* = .10, ŋ_p_^2^ = .03.

The repeated measures MANCOVA, controlling for the effects of age and treatment length, yielded no significant interaction effect between time and height deviation groups at after 1-year of treatment in patient, Pillai’s trace = .054, *F*_(5, 40)_ = .458, *p* = .80, ŋ_p_^2^ = .054, and in parent report, Pillai’s trace = .023, *F*_(7, 84)_ = .278, *p* = .96, ŋ_p_^2^ = .023. Also the univariate repeated measures ANCOVA for the QoLISSY *total score*, revealed no significant interaction effect between time and height deviation groups after 1-year of treatment in child, *F*_(1, 47)_ = 0.00, *p* = .92, ŋ_p_^2^ = .00, and parent report, *F*_(1, 112)_ = .624, *p* = 0.431, ŋ_p_^2^ = .00, while controlling for age at baseline and treatment length. Univariate analysis are presented in Table 8.

**Table 8:** HRQOL changes from baseline (T0) and 1-year follow-up (T1) between patients who reached normal height and patients with current short stature after 1-year of treatment

		Short stature (≤ −2 *SDS*)	Normal height (> −2 *SDS*)			
Patient-reports		*M (SD)* (*N* = 28)	*M (SD)* (*N* = 20)	*F*	*p*	Ŋ_p_^2^
Physical	T0	59.38 (24.63)	51.88 (29.79)	1.137	.29	.02
	T1	61.61 (20.95)	63.92 (29.16)			
Social	T0	51.51 (23.33)	53.48 (31.68)	.075	.78	.00
	T1	58.04 (25.29)	63.86 (25.16)			
Emotional	T0	55.72 (28.72)	54.38 (27.59)	.024	.87	.00
	T1	61.90 (22.93)	60.33 (27.59)			
Coping	T0	61.25 (20.05)	64.25 (25.59)	.00	.94	.00
	T1	57.68 (17.36)	60.68 (17.06)			
Beliefs	T0	52.46 (36.06)	50.00 (30.28)	.03	.85	.00
	T1	50.00 (32.59)	49.38 (34.11)			
Total score ^a^	T0	55.42 (22.94)	55.16 (28.06)	0.10	.92	.00
	T1	61.15 (21.17)	61.24 (26.34)			
Parents-reports		*M (SD)* (*N* = 41)	*M (SD)* (*N* = 53)	*F*	*p*	Ŋ_p_^2^
Physical	T0	54.96 (24.11)	47.88 (27.56)	.65	.41	.00
	T1	57.22 (24.03)	55.72 (27.00)			
Social	T0	52.68 (24.44)	49.95 (25.12)	.01	.90	.00
	T1	57.58 (24.58)	56.32 (27.49)			
Emotional	T0	51.26 (25.17)	50.41 (23.96)	.25	.61	.00
	T1	56.33 (25.13)	58.37 (25.52)			
Coping	T0	52.57 (20.60)	50.10 (15.67)	.19	.66	.00
	T1	54.37 (18.18)	50.07 (16.53)			
Beliefs	T0	57.93 (29.94)	50.71 (29.99)	.00	.97	.00
	T1	57.01 (31.78)	50.71 (29.38)			
Future	T0	62.32 (28.33)	52.74 (30.96)	.64	.42	.00
	T1	64.27 (26.77)	59.06 (29.94)			
Effects on parents	T0	55.62 (23.75)	49.11 (22.06)	.06	.79	.00
	T1	58.94 (21.70)	54.70 (24.18)			
Total score ^a^	T0	55.00 (22.78)	50.61 (23.73)	.62	.43	.00
	T1	58.88 (22.09)	57.82 (24.70)			

Note: Analysis: Univariate analyses of covariance. *M* = Mean, *SD* = Standard deviation, *F*=F-value, *p* = P-value, Ŋp2 = partial eta square, T0 = baseline, T1 = 12 month after start of hGH treatment

^a^ sum of the scales physical, social, emotional

Finally, the analysis of the relationship between height change and the HRQOL change prove significant positive correlation between height SDS increase (height difference between T1 and baseline) and total HRQOL gain (difference between QoLISSY *total score* at T1 and at baseline) in both the child (*r* = − 0.38, *p* ≤ 0.01) and parent-reports (*r* = − 0.18, *p* = 0.04). This suggests that a larger increase in body height is associated with greater improvement in the total HRQOL which reflects physical, social and emotional HRQOL.

## Discussion

Among the different health outcomes that can be measured, HRQOL has become a universally recognized endpoint to assess because of the potential of HRQOL instruments to reflect treatment benefits that affects patients actual value [7]. However, for rare conditions such endocrine short stature, use of generic HRQOL instruments is limited. Although generic instruments are well-validated and allow for comparison across populations, they often fail to detect small, but clinically significant changes over time in HRQOL due to the absence of disease-specific aspect of affected patients’ lives that have an impact on their HRQOL [7]. This aspect is supported by the results of this study. While the generic KIDSCREEN instrument is not able to detect changes in HRQOL in this sample, the disease-specific QoLISSY questionnaire detects changes of HRQOL within the course of hGH treatment between the treated and the untreated group in the patient-report. Treated children/adolescents who are diagnosed with IGHD or SGA reported a significant increase of *physical*, *social* and *emotional* HRQOL from baseline assessment throughout one year of treatment, while untreated patients of the control group diagnosed with ISS reported a decrease in these HRQOL domains over time. However, effect sizes for these results were rather small. Although differences were not statistically significant in parent-report, the results demonstrate a trend that parents of children who were treated with hGH reported an increase in their children’s HRQOL, while parents of children who were not treated reported a decrease in most domains throughout treatment. Besides, results of the correlation analysis between changes in the QoLISSY *total score* and height SDS gain shows that increased height results in improved physical, social and emotional HRQOL.

Similar outcomes were found in studies, showing that disease-specific instruments are able to detect changes in HRQOL in populations affected by rare health conditions. Lem et al. (2012) also revealed, that only the disease-specific instrument (TACQOL-S) showed improvement in HRQOL in treated children with hGH compared to untreated children, while the generic instrument did not reveal any HRQOL changes. Thus, when measuring the impact of treatment and to capture the change over time, a psychometric solid and disease-specific instrument is needed [7, 48].

Results of the repeated measures MANCOVA testing the HRQOL changes from baseline and 1-year follow-up (T1) between patients who reached normal height and patients with current short stature at T1 showed no significant results in the current study. This might indicate that reaching a normal height, either because of treatment or because of normal development, did not have a significant effect on the overall HRQOL or any of its specific domains. Although the QoLISSY questionnaire was not able to detect these longitudinal HRQOL changes from baseline to 1-year after treatment between patients who reached normal height and patients with current short stature after treatment (see Table 8), the questionnaire was able to detect differences between height deviation groups at baseline (Table 6 – social HRQOL in self- and parent-report). On the one hand, this might indicate, that it is very likely that the change in HRQOL from baseline to 1-year after treatment is not depending on the treatment or the increase in height, on the other hand, the questionnaire might lack sensitivity to detect clinically significant changes and that further studies are necessary to clarify this issue.

In addition to the debate between using generic or disease-specific HRQOL instruments, another aspect under discussion is the benefits of using self-reports vs. proxy-reports (ex. parents) when assessing HRQOL in pediatric populations. Self-reports are generally the preferred source of HRQOL data because of the concern of inaccurate reporting by proxies [12, 49]. However, when comparing the reported data between the child- and parent-report of the QoLISSY instrument in this study, analyses of ICC values indicated moderate parent-child agreement, except for the *coping* and *treatment* subscale, where the highest disagreement was observed. These findings underline, that especially psychosocial aspects of children’s HRQOL (such as coping strategies) cannot be observed and reported by a proxy as reliably as more obvious aspects (e.g. physical aspects) [12, 50, 51]. Thus, this study provides on the one hand support to use both self- and observed-reported HRQOL data in order to gain a comprehensive and more complementary view of the child’s experience of illness. On the other hand, when using child reported data in longitudinal studies, the cognitive development process of the children [52] needs to be considered. Opinions, feelings and attitudes might change quickly resulting in score changes that influences the responsiveness when assessing HRQOL. Hence, Coq et al. (2000) recommended the use of parent-reports in longitudinal intervention studies [53]. Also in this study, cognitive development changes might have influenced the responsiveness positively, since the child-report yielded significant changes over time in HRQOL, while the parent-report did not.

Internal consistency, as assessed by Cronbach’s alpha, was satisfactory in all scales with *α* > .70, except for the *coping* scale at T1 in child-report (*α =* .65). The Cronbach’s alpha values found in this study were similar to those observed in the original QoLISSY study [33], with the exception of the *coping* scale in the child-report, in which α was > .70 in the original study. A possible explanation for the inadequate Cronbach alpha value for the *coping* scale might be that the item content of this scale may be irrelevant or not correctly portray the experience of a patient population that receives treatment because of their short stature, thus leading to inconsistent responses. Furthermore, the lower Cronbach’s alpha value might suggest a poorer interrelatedness between the items of this scale, reflecting that coping is a heterogeneous construct, consisting of many facets and various coping strategies that are difficult to capture within one scale. Besides, the lower value might reflect difficulties in understanding item wordings, which might have let to inconsistent responses.

Also the scale inter-correlation values of the QoLISSY questionnaire were very similar to the values found in the original development and validation study of the QoLISSY questionnaire [9], including nonsignificant or low inter-correlations of the *coping* and *treatment* scales with all other scales of the QoLISSY instrument. With the exception of a few scales, most QoLISSY scales showed significant moderate correlations (*r* > .30) with the KIDSCREEN-10 Index, supporting the criterion validity of the instrument and that both instruments measure the construct of HRQOL. Nevertheless, the moderate correlations also indicate towards a discrepancy between both measures, which further supports the importance of using disease-specific instruments that are able to catch unique aspects experienced by the affected people that generic instruments would miss.

Comparisons of HRQOL scores between participants that were treated with hGH compared to participants that were not treated demonstrated known-group validity for the *total score* in child- and parent-report, with children who were about to start treatment and parents of these children reported a lower HRQOL, compared to children who remained untreated at baseline. After treatment, no significant differences in HRQOL between the treated and the untreated sample were found in child- and parent-reports. Contradictory to results of known-group validity analysis found in the original development and validation study of the QoLISSY questionnaire [47], QoLISSY was not able to distinguish between the severity level of short stature (height SDS) at both points of measurement in the current sample. This might be due to the fact, that at baseline most children in the sample were short statured (height SDS ≤ − 2). Furthermore, this study was designed as a longitudinal study and thus a cross-sectional analysis as conducted for the known-group validity might be not as appropriate as a responsiveness analysis that combines both measurement points. As already discussed above, QoLISSY proves to be able to detect changes in child reported HRQOL within the course of treatment.

One limitation within this study is that a confirmatory factor analysis was not calculated because of the small sample size. Nevertheless, the factor structure of the QoLISSY instrument was previously ascertained for identifying the final scale structure of the instrument. The initial confirmatory factor analysis produced the three core quality of life domains (physical, social, emotional) which composed to the QoLISSY *total score*, while the other domains were used as determinants [33]. In general, the smaller sample size and drop-out rate might have biased the results. However, keeping in mind that endocrine short stature is a rare disease, this study included a relatively large sample size. Still, the sample is very selective because all of them contacted growth clinics seeking for treatment options. Therefore, it can be assumed that the sample is highly motivated and not necessarily representative of the overall target population. Since this study was an observational study based on “natural” treated and untreated groups complying with standard clinical practice and appropriate prescribing guidelines (determined by European Medicine Agency), we were not able to include a normal statured, age-matched not treated control group with IGHD or SGA for comparison as this would be considered unethical. Furthermore, the sample description (Table 1) shows that some patients, especially patients in the control group with the diagnosis ISS, already reached a normal height by definition (< − 2 *SDS*) at baseline. We used the common German reference values of Kromeyer-Hausschild et al. (2001) to calculate the height SDS, which might resulted in slightly different scores than the clinician used when diagnosing the patient. Thus, the sample included also a few patients who had normal height at baseline, which might have biased the results. In general, significant results should be interpreted with caution because of the small sample and small effect sizes. Another point is, that due to the design of the study our responsiveness analyses were limited to 12 months of treatment. However, greater changes in HRQOL might also appear after some time having finished the treatment.

## Conclusions

These psychometric analyses of the QoLISSY questionnaire support the reliability, validity, and ability of the instrument to detect change over time and thus support its usefulness to measure HRQOL in a pediatric endocrine population receiving hGH treatment. Hence, in addition to clinical endpoints, HRQOL can also be adequately assessed within a clinical setting. Further use of the QoLISSY in longitudinal studies may provide greater insight into the clinical meaningfulness of changes in HRQOL scores.

## Abbreviations

hGH: Human Growth Hormone
HRQOL: Health-related Quality of Life
IGHD: Idiopathic Growth Hormone Deficiency
ISS: Idiopathic Short Stature
PRO: Patient-reported outcome
QoLISSY: Quality of Life in Short Stature Youth
SDS: Standard Deviation Score

## References

[1] Eiser C, Jenney M (2007). Measuring quality of life. Arch Dis Child.

[2] Brütt AL, Sandberg DE, Chaplin J, Wollmann H, Noeker M, Koltowska-Haggstrom M (2009). Assessment of health-related quality of life and patient satisfaction in children and adolescents with growth hormone deficiency or idiopathic short stature - part 1: a critical evaluation of available tools. Horm Res.

[3] Bullinger M, Quitmann J, Power M, Herdman M, Mimoun E, DeBusk K (2013). Assessing the quality of life of health-referred children and adolescents with short stature: development and psychometric testing of the QoLISSY instrument. Health Qual Life Outcomes.

[4] Varni JW, Burwinkle TM, Katz ER, Meeske K, Dickinson P (2002). The PedsQL in pediatric cancer: reliability and validity of the pediatric quality of life inventory generic Core scales, multidimensional fatigue scale, and Cancer module. Cancer.

[5] Chen TH, Li L, Kochen MM (2005). A systematic review: how to choose appropriate health-related quality of life (HRQOL) measures in routine general practice?. The Journal of Zhejiang University Science B: Biomedicine & Biotechnology.

[6] Bingham CO, Noonan VK, Auger C, Feldmann DE, Ahmed S, Barlett SJ (2017). Montreal accord on patient-reported outcomes use series - paper 4: patient-reported outcomes can infrom clinical decision making in chronic care. J Clin Epidemiol.

[7] Rüther A, Wang-Rieger D, Elstein D, Guyatt G (2016). Aspects of patient reported outcomes in rare diseases: a discussion paper. Int J Technol Assess Health Care.

[8] Bullinger M (2002). Assessing health related quality of life in medicine. An overview over concepts, methods and applications in international research. Restor Neurol Neurosci.

[9] Versteegh M, Leunis A, Uyl-de Groot C, Stolk E (2012). Condition-specific preference-based measures: benefit or burden?. Value Health.

[10] Price VE, Klaassen RJ, Bolton-Maggs PH, Grainger JD, Curtis C, Wakefield C, et al. Measuring disease-specific quality of life in rare populations: a practical approach to cross-cultural translation. Health Qual Life Outcomes. 2009;7. 10.1186/1477-7525-7-92 PMC2773763 19852803

[11] Kaplan RM, Ries AL (2007). Quality of life: concept and definition. COPD: J Chron Obstruct Pulmon Dis.

[12] Quitmann J, Rohenkohl A, Sommer R, Bullinger M, Silva N. Explaining parent-child (dis)agreement in generic and short stature-specific health related quality of life reports: do family and social relationships matter? Health Qual Life Outcomes. 2016;14. 10.1186/s12955-016-0553-0 PMC5075198 27769269

[13] Wu HH, Li H, Gao Q (2013). Psychometric properties of the Chinese version of the pediatric quality of life inventory 4.0 generic core scales among children with short stature. Health Qual Life Outcomes.

[14] Stephen MD, Varni JW, Limbers CA, Yafi M, Heptulla RA, Renukuntla VS (2011). Health-related quality of life and cognitive functioning in pediatric short stature: comparison of growth-hormone-naive, growth-hormone-treated, and healthy samples. Eur J Pediatr.

[15] Theunissen NC, Kamp GA, Koopman HM, Zwinderman KA, Vogels T, Wit JM (2002). Quality of life and self-esteem in children treated for idiopathic short stature. J Pediatr.

[16] Xu X, Wen J, Peng DX, Liu Y (2013). Quality of life in children with short stature: an analysis using PedsQL. Zhongguo dang dai er ke za zhi = Chinese journal of contemporary pediatrics.

[17] Lem A, Jobse I, van Der Kaay D, de Ridder M, Raat H, Hokken-Koelega A (2012). Health-related quality of life in short children born small for gestational age: effects of growth hormone treatment and postponement of puberty. Horm Res Paediatr.

[18] Wit JM, Clayton PE, Rogol AD, Savage MO, Saenger PH, Cohen P (2008). Idiopathic short stature: definition, epidemiology, and diagnostic evaluation. Growth Hormon IGF Res.

[19] Barstow C, Rerucha C (2015). Evaluation of short and tall stature in children. Am Fam Physician.

[20] Abe S, Okumura A, Mukae T, Nakazawa T, Niijima S, Yamashiro Y (2009). Depressive tendency in children with growth hormone deficiency. J Paediatr Child Health.

[21] Silva N, Bullinger M, Sommer R, Rohenkohl A, Witt S, Quitmann J (2018). Children's psychosocial functioning and parents' quality of life in paediatric short stature: the mediating role of caregiving stress. Clin Psychol Psychother.

[22] Carel J, Chatelain P, Rochiccioli P, Chaussain J (2003). Improvement in adult height after growth hormone treatment in adolescents with short stature born small for gestational age: results of a randomized controlled study. J Clin Endocrinol Metab.

[23] Finkelstein B, Imperiale T, Speroff T, Marrero U, Radcliffe D, Cuttler L (2002). Effect of growth hormone therapy on height in children with idiopatic short stature. Arch Pediatr Adolesc Med.

[24] Ranke MB, Lindberg A (2010). Obeserved and predicted growth responses in prepubertal children with growth disorders: guidance of growth hormone treatment by empirical variables. J Clin Endocrinol Metab.

[25] Wit JM, Reiter EO, Ross JL, Saenger PH, Savage MO, Rogol AD (2008). Idiopathic short stature: management and growth hormone treatment. Growth Hormon IGF Res.

[26] Bullinger M, Koltowska-Haggstrom M, Sandberg D, Chaplin J, Wollmann H, Noeker M (2009). Health-related quality of life of children and adolescents with growth hormone deficiency or idiopathic short stature - part 2: available results and future directions. Horm Res.

[27] Geisler A, Lass N, Reinsch N, Uysal Y, Singer V, Ravens-Sieberer U (2012). Quality of life in children and adolescents with growth hormone deficiency: association with growth hormone treatment. Horm Res Paediatr.

[28] Schena L, Meazza C, Pagani S, Paganelli V, Bozzola E, Tinelli C (2017). Efficacy of long-term growth hormone therapy in short non-growth hormone-deficient children. J Pediatr Endocrinol Metab.

[29] Ahmid M, Perry CG, Ahmed SF, Shaikh MG (2016). Growth hormone deficiency during young adulthood and the benefits of growth hormone replacement. Endocrine Connections.

[30] Cole TJ (1990). The LMS method for constructing normalized growth standards. Eur J Clin Nutr.

[31] Kromeyer-Hausschild K, Wabitsch M, Kunze D, Geller F, Geiß H, Hesse V (2001). Perzentile für den Body-mass-Index für das Kindes- und Jugendalter unter Heranziehung verschiedener deutsche Stichproben. Monatsschr Kinderheilkd.

[32] FDA. Guidance for Industry Patient-Reported Outcome Measures: Use in Medical Product Development to Support Labeling Claims. Silver Spring, MD: Food and Drug Administration, 2009.

[33] The European QoLISSY Group. Quality of life in short stature youth. The QoLISSY questionnaire – User's manual. Lengerich: Pabst Science Publishers; 2013.

[34] Bullinger M, Sommer R, Pleil A, Mauras N, Ross J, Newfield R, et al. Evaluation of the American-English quality of life in short stature youth (QoLISSY) questionnaire in the United States. Health Qual Life Outcomes. 2015;13. 10.1186/s12955-015-0236-2 PMC4424504 25889818

[35] Quitmann J, Giammarco A, Maghnie M, Napoli F, Di Giovanni I, Carducci C (2017). Validation of the Italian quality of life in short stature youth (QoLISSY) questionnaire. J Endocrinol Investig.

[36] Rohenkohl A, De Schepper J, Vanderfaeillie J, Fricke K, Hendrickx S, Lagrou K (2014). Validation of the Flemish version of the quality of life in short stature youth (QoLISSY) questionnaire. Acta Clin Belg.

[37] Rohenkohl A, Stalman S, Kamp G, Bullinger M, Quitmann J (2016). Psychometric performance of the quality of life in short stature youth (QoLISSY) questionnaire in the Netherlands. Eur J Pediatr.

[38] Rohenkohl A, Sommer R, Bestges S, Kahrs S, Klingebiel KH, Bullinger M (2015). Leben mit Achondroplasie: Wie beurteilen junge Menschen mit disproportioniertem Kleinwuchs ihre Lebensqualität und mit welchen Faktoren ist sie assoziiert?. Zeitschrift für Kinder- und Jugendpsychiatrie und Psychotherapie.

[39] Sommer R, Blömeke J, Bullinger M, Quitmann J (2018). The psychometric evaluation of the quality of life in short stature youth (QoLISSY) instrument for German children born small for gestational age. Journal of Endcrinological Investigation.

[40] The KIDSCREEN Group Europe. The KIDSCREEN questionnaires: quality of life for children and adolescents – handbook. Lengrich: Pabst Science Publishers; 2006.

[41] Silva N, Bullinger M, Quitmann J, Ravens-Sieberer U, Rohenkohl A (2013). HRQoL of European children and adolescents with short stature as assessed with generic (KIDSCREEN) and chronic-generic (DISABKIDS) instruments. Expert Rev Pharmacoecon Outcomes Res.

[42] Corp IBM (2012). IBM SPSS statistics for windows. 21.0 ed.

[43] Terwee CB, Bot SD, de Boer MR, van der Windt DA, Knol DL, Dekker J (2007). Quality criteria were proposed for measurement properties of health status questionnaires. J Clin Epidemiol.

[44] Nunnally J (1994). Psychometric theory 3 ed.

[45] Sneeuw KC, Sprangers MA, Aaronson NK (2002). The role of health care providers and significant others in evaluating the quality of life of patients with chronic disease. J Clin Epidemiol.

[46] Landis JR, Koch GG (1977). Measurement of observer agreement for categorical data. Biometrics.

[47] Cohen J (1988). Statistical power analysis for the behavioral sciences.

[48] Guyatt GH, Bombardier C, Tugwell PX. Measuring disease-specific quality of life in clinical trials. Can Med Assoc J. 1986;134. PMC1490966 3955482

[49] Varni JW, Burwinkle TM, Lane MM. Health-related quality of life measurement in pediatric clinical practice: an appraisal and precept for future research and application. Health Qual Life Outcomes. 2005;3. 10.1186/1477-7525-3-34 PMC1156928 15904527

[50] Klassen AF, Miller A, Fine S (2005). Agreement between parent and child report of quality of life in children with attention-deficit/hyperactivity disorder. Child Care Health Dev.

[51] Eiser C, Morse R (2001). Quality-of-life measures in chronic diseases of childhood. Health Technol Assess.

[52] Oerter R, Dreher E. Jugendalter: Kognitive Entwicklung. In: Oerter R, Montada L, editors. Entwicklungspsychologie. Beltz Verlage Weinheim, Basel, Berlin: Beltz Verlage; 2002. p. 273–6.

[53] le Coq EM, Boeke AJP, Bezemer PD, Colland VT, van Eijk JTM (2000). Which source should we use to measure quality of life in children with asthma: the children themselves or their parents?. Qual Life Res.

